# Supplementary material for: Novel Plant-Protein (Quinoa) Derived Bioactive Peptides with Potential Anti-Hypercholesterolemic Activities: Identification, Characterization and Molecular Docking of Bioactive Peptides
Source: Foods. 2023 Mar 20;12(6):1327. doi: 10.3390/foods12061327 (PMC10048307; doi:10.3390/foods12061327)
Supplement: Supplementary file 1 [file foods-12-01327-s001.zip › foods-2122383-supplementary.pdf]

Table S1. List of peptides identified in chymotrypsin hydrolysed quinoa protein hydrolysate QC6

Peptide	Peptide Ranker score
AAVHAF	0.438779
AGLR	0.544331
AHCGGLPY	0.689212
ALDSPGSR	0.462201
ALSKR	0.196289
ANPALAVGY	0.455945
ASNLDNPSPEGTVM	0.521523
AVGVI	0.122801
CALVGL	0.507875
CDCP	0.887083
CVLSPL	0.509805
CVSP	0.329948
CYTF	0.853922
DAVLGV	0.128426
DFLM	0.915466
DFTF	0.920428
DGPVR	0.432235
DH	0.147981
DVTSPL	0.243089
DYAVSF	0.375715
EGGPVG	0.27601
ELLL	0.208407
ESTP	0.087021
EVLP	0.112021
EVTSGDT	0.0412863
FE	0.589707
FEVEAPGNPVVM	0.36185
FFE	0.934729
FSAGGLP	0.808746
FTVM	0.537171
GAGAI	0.365362
GARR	0.523569
GCTSR	0.289798
GERFAVNPV	0.290352
GFVDLLRDDY	0.376191
GLFTEDR	0.246168
GPLTR	0.487858
GSTASSCS	0.164599
GVGR	0.345362

GVLVG	0.16568
HD	0.128192
HDSF	0.573106
HLAR	0.243625
HMLH	0.509564
HMPALTEIFGDDSVL	0.262311
HR	0.331511
HSHVH	0.133601
HVAF	0.448191
HVASGAGPW	0.543078
HVQGHPALPGVPAHW	0.555346
KEAPAL	0.224453
KGDCHPTH	0.441731
KIGGIGTVPVGRVE	0.177007
KIVLDSDDPLFGGF	0.706601
KLKVDH	0.0722252
KPGGTAGSALPRPAHW	0.608191
KR	0.213072
KTPAL	0.267865
KTTLAK	0.0544679
LADSPH	0.261273
LDLP	0.484448
LKFT	0.368847
LLPYH	0.533626
LLTGVL	0.190991
LLVG	0.236174
LPLLR	0.613775
LR	0.569984
LRL	0.564172
LV	0.0991475
LVDLR	0.195511
LVGGTGEEGEAL	0.101911
LVPPK	0.33989
LYLLLARSM	0.345476
LYNDWDLR	0.679917
LYVTVPMHLVVM	0.267707
ME	0.198233
MFVPVPH	0.55808
ML	0.894564
MLLALAGNQPL	0.381893
MLLL	0.756994
MNAGV	0.220838
MNAR	0.374396
MPQGT	0.494047
MSSL	0.414735

MTTSPK	0.11994
MVLP	0.52787
MYLY	0.748653
NLSKLL	0.36374
NRGT	0.209343
PEPTIDE	0.111635
PGGAR	0.640441
PSSKR	0.245177
PYTTQSP	0.129124
QGVLEGVELCR	0.321682
QHPHGLGALCAAPPST	0.720324
QKSPNVTGLTPVL	0.409057
QLNAH	0.159603
QLSPAVPPAET	0.292052
RAGR	0.417105
RFTWVT	0.469549
RGEFAVFY	0.455717
RLEDLRIPVAY	0.192841
RR	0.565498
RSQTGKADPT	0.0910118
SELLK	0.131421
SGPAR	0.632577
SPLEAGR	0.349217
SVGMDAVFY	0.464034
TAAPR	0.226615
TAEKDEST	0.0334789
TDGLTSLDRY	0.131845
TELP	0.111454
TFVLPPTELN	0.179309
TGATVLCAPRKN	0.243014
THTLLLLP	0.276065
TLPEDLTAP	0.127875
TLPGR	0.413895
TREVVPGMIVTGMEVAEIDGAPR	0.0501995
TTSP	0.0873582
TYVF	0.398271
VAEGH	0.0805037
VDPEDLLR	0.395537
VGDPR	0.376834
VGGR	0.389969
VGGV	0.179893
VLASEGGPGL	0.340721
VLGT	0.120896
VLLPR	0.333531
VPISGFEGDNMIER	0.461132

VPISGFEGDNMIERSTNL	0.322682
VSAP	0.171359
VSVAH	0.0687475
VTVH	0.0362087
VYML	0.567064
WEGTSVVNP	0.122929
YATL	0.237529

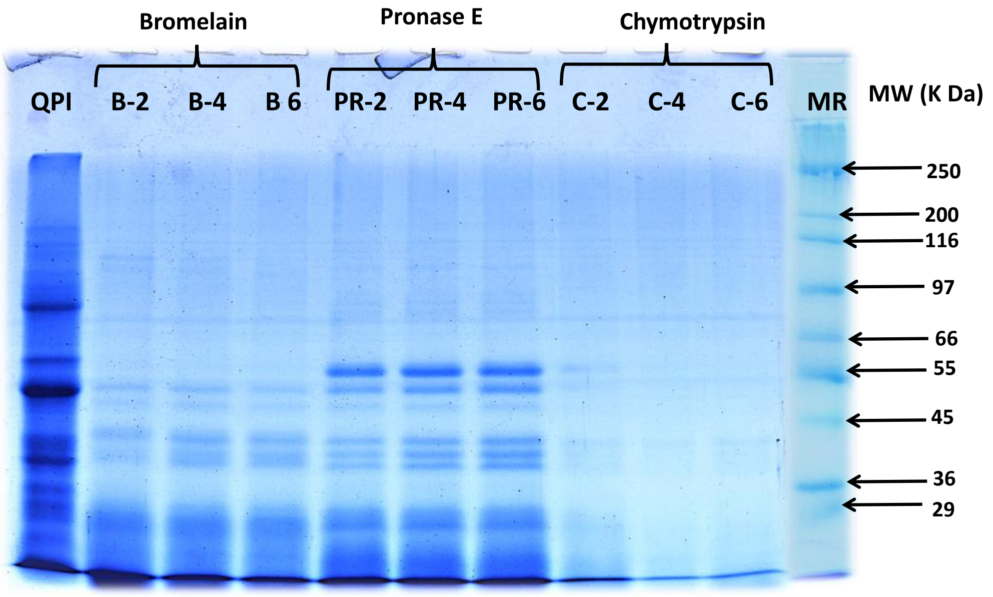

Figure S1. Electrophoretic patters of quinoa proteins and quinoa protein hydrolysates.

Keynotes: B-2, B-4, B6, PR-2, PR-4, PR-6 and C-2, C-4 and C-6 represents Bromelain, Pronase E and Chymotrypsin generated hydrolysates after 2, 4 and 6 h of hydrolysis, MR: wide range protein marker, K Da: Kilo Dalton. **Source**: Mudgil et al. (2020) with permission to reproduce figure obtained from Elsevier via License no: 5487580410082)
